# Left Cathodal Trans-Cranial Direct Current Stimulation of the Parietal Cortex Leads to an Asymmetrical Modulation of the Vestibular-Ocular Reflex^☆^

**DOI:** 10.1016/j.brs.2013.07.002

**Published:** 2014-01

**Authors:** Qadeer Arshad, Yuliya Nigmatullina, R. Edward Roberts, Vamsee Bhrugubanda, Paladd Asavarut, Adolfo M. Bronstein

**Affiliations:** Department of Nero-otology, Division of Brain Sciences, Charing Cross Hospital Campus, Imperial College London, Fulham Palace Road, London W6 8RF, UK

**Keywords:** Vestibular cortical processing, tDCS, Parietal balance, Vestibular-ocular reflex

## Abstract

Multi-sensory visuo-vestibular cortical areas within the parietal lobe are important for spatial orientation and possibly for descending modulation of the vestibular-ocular reflex (VOR). Functional imaging and lesion studies suggest that vestibular cortical processing is localized primarily in the non-dominant parietal lobe. However, the role of inter-hemispheric parietal balance in vestibular processing is poorly understood. Therefore, we tested whether experimentally induced asymmetries in right versus left parietal excitability would modulate vestibular function. VOR function was assessed in right-handed normal subjects during caloric ear irrigation (30 °C), before and after trans-cranial direct current stimulation (tDCS) was applied bilaterally over the parietal cortex. Bilateral tDCS with the anode over the right and the cathode over the left parietal region resulted in significant asymmetrical modulation of the VOR, with highly suppressed responses during the right caloric irrigation (i.e. rightward slow phase nystagmus). In contrast, we observed no VOR modulation during either cathodal stimulation of the right parietal cortex or SHAM tDCS conditions. Application of unilateral tDCS revealed that the left cathodal stimulation was critical in inducing the observed modulation of the VOR. We show that disruption of parietal inter-hemispheric balance can induce asymmetries in vestibular function. This is the first report using neuromodulation to show right hemisphere dominance for vestibular cortical processing.

## Introduction

The insular-parietal cortical region is known to be involved in vestibular cortical processing based on data from functional imaging and lesion studies [1], [2], however the effect of disrupting inter-hemispheric parietal balance [3] upon vestibular processing remains unknown. Trans-cranial direct current stimulation (tDCS) has previously been shown to modulate cortical excitability through the induction of transient changes in local field polarity [3], [4]. Moreover, concurrent application of opposite polarity stimulation over parietal regions has been reported to alter the parietal balance between the two hemispheres [4], [5].

Normal vestibular responses, as assessed by the vestibular-ocular reflex (VOR), elicited by vestibular activation are approximately symmetrical, irrespective of whether the right or left labyrinth is stimulated. However, the VOR is plastic in that it can be bi-directionally modified by both visual and non-visual input (i.e. VOR suppression is observed if subjects focus on a real or imagined target) [6].

Functionally, the VOR is critical for gaze stabilization during head perturbations, and is mediated by a combination of vestibular and retinal velocity signals. Despite the significant involvement of brainstem centers in the VOR, higher order integration of visuo-vestibular signals may be critical for the conscious perception of body position in space and potentially to regulate reflexes such as the VOR [7]. Moreover, in a recent study an asymmetrical handedness-related down-regulation of the VOR was demonstrated as a result of viewing bistable perceptual visual stimuli (i.e. binocular rivalry) or performing a visualized spatial attentional task during concurrent vestibular stimulation. It was proposed that the effect occurred as a result of engaging overlapping cortical parietal networks in the non-dominant hemisphere resulting in disruption of parietal hemispheric balance [8]. Hence, we sought to modulate relative excitability levels in the parietal lobes of left and right hemispheres with tDCS, thereby inducing parietal lobe imbalance, in order to assess the effect upon the VOR.

## Materials and methods

### Subjects

In total twenty right-handed subjects (14 male, mean age 25.6 years, range 19–35) without any brain stimulation contra-indications, nor history of labyrinthine or neurological disorder and naive to the purpose of the study gave written consent to take part in the study as directed by the local ethics research committee The 20 subjects were equally split into two groups. The first set (8 males) participated in the bilateral tDCS experiment whilst the fellow set of 10 subjects (6 males) took part in the unilateral tDCS experiment.

### Trans-cranial direct current stimulation (tDCS)

Stimulation was applied using a battery driven stimulator (neuroConn GMBH, ilmenau, Germany). The current had a ramp up time of 10 s at which point a constant current of an intensity of 1.5 mA was applied for a total duration of 15 min, after which the current ramped down in a 10 s fade out period, in line with current safety guidelines [9].

#### Bilateral tDCS stimulation

**Figure 1:**
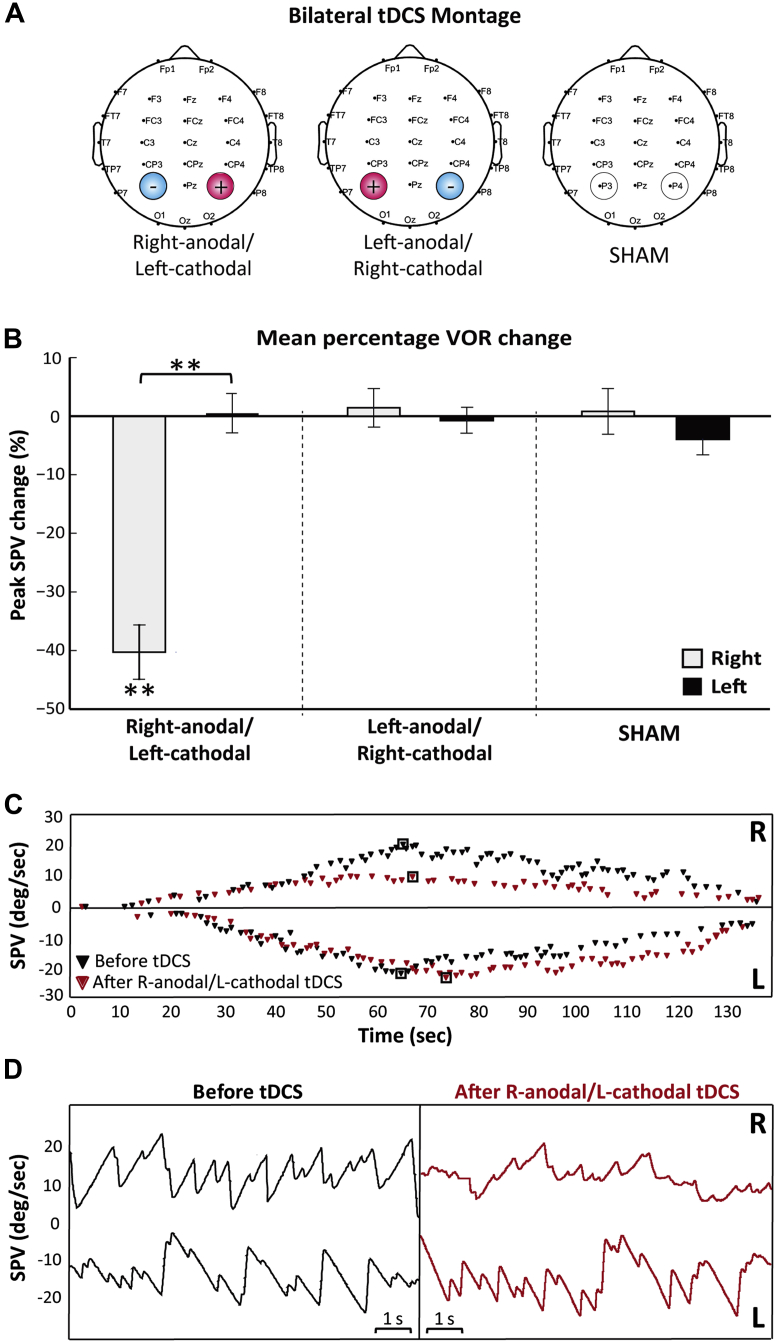
Bilateral tDCS montage and the effect of bilateral tDCS stimulation on the VOR. A. The electrodes delivering tDCS were placed on the left and right parietal cortex, over P3 and P4 respectively (10–20 EEG international positioning system). Three types of bilateral parietal tDCS stimulation were applied: anodal over right hemisphere and cathodal over left hemisphere (referred to as Right-anodal/Left-cathodal), cathodal over right hemisphere and anodal over left hemisphere (referred to as Left-anodal/Right-cathodal) and SHAM. B. Right-anodal/left-cathodal resulted in asymmetrical modulation of the VOR with significant decrease in peak slow phase velocity (SPV) during the right caloric with no significant change seen during the left caloric. Left-anodal/right-cathodal and SHAM stimulations resulted in symmetrical VOR responses with no modulation of the peak SPV during right or left calorics. Data marked ** are significant at *P* < 0.01. C. VOR responses from a single subject are shown over time for the right (R; positive SPV) and left (L; negative SPV) calorics before (black diamonds) and after (red diamonds) Right-anodal/Left-cathodal stimulation over the parietal cortex. The black boxes indicate the peak SPV. D. Oculomotor responses are shown over 10 s around the peak SPV (from C) for right (R) and left (L) calorics before (black) and after (red) tDCS right-anodal/left-cathodal stimulation. Significant reduction in oculomotor SPV is seen during the right caloric after right-anodal/left-cathodal parietal stimulation. (For interpretation of the references to color in this figure legend, the reader is referred to the web version of this article.)

For anodal stimulation of the right parietal cortex the anodal electrode was placed over P4 (international 10–20 system for EEG electrode placement, area 25 cm^2^), whilst the cathode (area 25 cm^2^) was placed over the left P3 (right-anodal/left-cathodal condition). Reciprocally, anodal stimulation of the left parietal cortex involved the anodal electrode placed over the left P3 and the cathodal electrode placed over the right P4 (left-anodal/right-cathodal condition). This montage has previously been shown to be successful in inducing parietal asymmetries [5]. For the SHAM condition the electrodes were placed over the same target areas as in the tDCS condition (right anodal over P4) (Fig. 1A). However, in the SHAM stimulation condition the stimulator was ramped down after 30 s ensuring that the initial sensation of the tDCS and SHAM condition did not differ, but without providing any actual stimulation.

#### Unilateral tDCS stimulation

**Figure 2:**
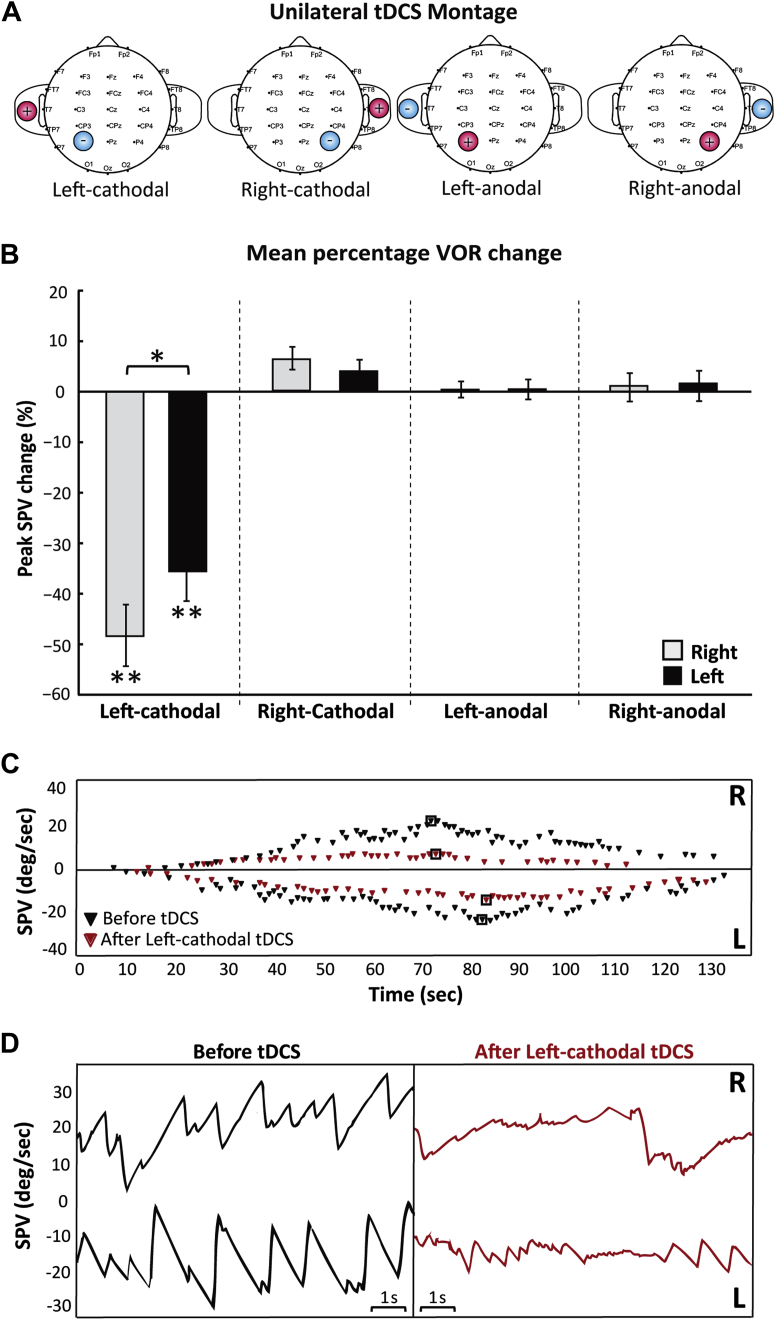
Unilateral tDCS montage and the effect of unilateral tDCS stimulation on the VOR. A. Electrode montage for the 4 different conditions (Left-cathodal, Right-cathodal, Left-anodal, Right-anodal). The reference electrode was placed on the ipsilateral shoulder. B. Left-cathodal stimulation shows a bilateral reduction albeit a significantly greater reduction for right caloric stimulation. Data marked * are significant at *P* < 0.05, ** are significant at *P* < 0.01. C. VOR responses from a single subject are shown over time for the right (R; positive SPV) and left (L; negative SPV) calorics before (black diamonds) and after (red diamonds) Left-cathodal stimulation. The black boxes indicate the peak SPV. Oculomotor responses are shown over 10 s around the chosen peak SPV for right (R) and left (L) calorics before (black) and after (red) unilateral Left-cathodal stimulation. Significant reduction in oculomotor SPV is seen during the right and left caloric after left parietal cathodal stimulation. (For interpretation of the references to color in this figure legend, the reader is referred to the web version of this article.)

In order to separate the relative contributions of anodal and cathodal stimulation upon any potential modulation of the VOR by the bilateral tDCS stimulation (concurrent application of opposite polarity stimulation) we employed a unilateral tDCS condition. This involved application of either right-anodal or right-cathodal stimulation over P4, or left-anodal or left cathodal stimulation over P3 (i.e. right-anodal, right-cathodal, left-anodal, left-cathodal conditions; Fig. 2A). The reference electrode in all four conditions was placed on the ipsilateral shoulder (deltoid muscle). In order to reduce irritation and maximize subject comfort a larger reference electrode was used (area 35 cm^2^). Otherwise, the stimulus parameters for the tDCS were identical to that used for the bilateral stimulation condition.

### Vestibular stimulation

Following otoscopy, to exclude local contra-indications, subjects underwent caloric irrigation. The participants lay supine on a couch with the head tilted up 30° to obtain maximal activation of the horizontal semi-circular canals. The external auditory meatus was irrigated with water at 30 °C (cold water) at a rate of 500 ml/min for 40 s (CHARTR VNG; ICS medical) [10]. The onset of vertigo occurs approximately 20 s after the start of the irrigation reaching a peak at around 60 s. The total duration of the response lasted on average 3 min.

### Measurement of oculomotor response

In response to the caloric vestibular activation an oculomotor response (VOR in the form of ‘vestibular nystagmus’) is elicited: e.g. the right cold caloric results in leftward fast phase and rightward slow phase nystagmus and vice versa (Fig. 1D). Eye movements were recorded using a head mounted infra-red binocular video-oculography (VOG) system (CHARTR VNG; ICS medical). Analyses of eye movements were performed using a computerized automatic analysis programme (CHARTR VNG; ICS medical) that removes the quick phases (saccades) of the nystagmus and plots the slow phase velocity of the eye movement over a period of 100 s (rightward slow phase for right cold caloric and vice versa). The automated analysis programme required subjects to initially perform an eye movement calibration by following a pursuit target. Response intensity was determined by the peak slow phase eye velocity and the duration of the evoked vestibular nystagmus.

### Experimental procedure

#### Bilateral tDCS experiment

Each participant underwent 3 randomized sessions in accord with a latin square experimental design (right-anodal/left-cathodal, left-anodal/right-cathodal or SHAM), separated by a minimum of 4 days to minimize any potential tDCS carry over effects and any possibility of vestibular habituation. Each session began with two caloric tests (right and left ear randomized), in order to establish the pre-stimulation oculomotor response. The irrigations were separated by a period of 5 min to allow for after effects to subside. After the pre-stimulation caloric response, tDCS was applied for 15 min. Following tDCS, two further caloric irrigations (one per ear) were performed in order to obtain the post-tDCS caloric response. Thus, each subject was tested on 3 separate occasions and within each session each subject underwent 4 caloric irrigations (two per ear) in total.

#### Unilateral tDCS experiment

Each participant underwent 4 randomized sessions in accord with a latin square experimental design (right-anodal, right-cathodal, left-anodal or left-cathodal), with each condition separated by a minimum of 4 days. Otherwise, the experimental procedure was identical to that implemented in the bilateral tDCS experiment.

### Statistical analysis

#### Bilateral tDCS experiment

Percentage change in the peak of the slow phase velocity (SPV) of the VOR response was calculated for post-tDCS compared to baseline caloric SPV. The analysis was a within-subject repeated measures (ANOVA), which comprised the within-individual factors tDCS (right-anodal/left-cathodal, left-anodal/right-cathodal, SHAM); laterality of caloric (left, right) and time (before, after tDCS).

#### Unilateral tDCS experiment

We used the percentage change in baseline SPV to perform a within-subjects repeated measures ANOVA with factors tDCS (cathodal or anodal); laterality of caloric (left or right), and stimulation side (left or right).

For both stimulation conditions, post hoc tests were applied using Bonferroni corrections for multiple comparisons, differences were considered signiﬁcant at a level of *P* < 0.05. Sphericity in the ANOVA model was examined for using Mauchley's test. For non-spherical data the Greenhouse–Geisser correction was used. Statistical analyses were performed using SPSS 20.

## Results

Across all participants in both tDCS conditions and the SHAM condition the baseline peak VOR response for the caloric was symmetrical (i.e. right versus left) as expected (*P* > 0.05; paired *t*-test).

### Bilateral tDCS experiment

Following right-anodal/left-cathodal tDCS stimulation, we observed an asymmetrical modulation of the VOR. ANOVA with tDCS (three levels), time (two levels) and laterality of caloric (two levels) indicated a significant interaction between stimulation*laterality (*F*[1.4,12.6] = 10.9, *P* < 0.03), stimulation*time (*F*[1.2,10.6] = 13.8, *P* < 0.03) and laterality*time (*F*[1,9] = 11.4, *P* < 0.01). The interaction stimulation*laterality*time (*F*[1.2,11.1] = 41.8, *P* < 0.001) was most significant (Fig. 1B and C). Across all participants in both tDCS conditions and the SHAM condition the baseline peak VOR response for the caloric was symmetrical (i.e. right versus left) as expected (*P* > 0.05; paired *t*-test). Post hoc paired *t*-tests demonstrated a significant reduction in rightwards slow phase velocity (right caloric) following right-anodal/left-cathodal tDCS condition (*t*(9) = 6.2, *P* < 0.001, corrected; Fig. 1B). We observed this marked suppression of the VOR response in all 10 participants with a mean percentage reduction of 40% in the slow phase velocity (Fig. 1B, C, and D). There was no significant change in the VOR response attributable to the left caloric following right-anodal/left-cathodal parietal stimulation (*P* > 0.05 paired *t*-test).

The VOR response was symmetrical following either SHAM or left-anodal/right-cathodal tDCS stimulation (*P* > 0.05; paired *t*-test). In addition, in all conditions there was no effect upon the duration of the evoked vestibular nystagmus (data not shown). Thus, bilateral right-anodal/left-cathodal tDCS stimulation of the parietal cortex induced an asymmetrical VOR response with reduction of the rightward slow phase velocity following the right caloric (Fig. 1B, C, and D).

### Unilateral tDCS experiment

In the unilateral stimulation conditions, cathodal stimulation of the left parietal region (left-cathodal) induced a bilateral, though asymmetric, reduction of VOR response during caloric irrigation. ANOVA with tDCS (two levels), tDCS stimulation side (two levels) and laterality of caloric (two levels) showed that there was a significant main effect of tDCS type (*F*[1,7] = 32.9, *P* < 0.001) and stimulation side (*F*[1,7] = 30.2, *P* < 0.001). There was a significant interaction of tDCS type*stimulation side (*F*[1,7] = 57.5, *P* < 0.001), stimulation*laterality of caloric (*F*[1,7] = 9.49, *P* < 0.018) and tDCS type*stimulation side*laterality of caloric (*F*[1,7] = 38.4, *P* < 0.001). A post hoc paired *t*-test (2-tailed) showed a larger suppression of VOR response for the right caloric versus left caloric in left-cathodal condition (*t*(7) = −3.3, *P* = 0.013, Fig. 2B, C, D, E, and G). Thus, unilateral left-cathodal tDCS stimulation of the parietal cortex resulted in bilateral reduction of VOR responses following the right and the left caloric. This reduction was asymmetric with greater suppression of the rightward slow phase velocity (i.e. right caloric) with a mean reduction of 48% compared to the leftward slow phase velocity, which showed a mean reduction of 35% (i.e. left caloric).

Although not formally measured, all participants reported a subjective reduction in dizziness following the tDCS conditions which elicited a significant reduction in VOR response (i.e. in bilateral right-anodal/left-cathodal and unilateral left-cathodal conditions). In all the other stimulation conditions whereby no modulation of the VOR was observed, participants reported a similar intensity of dizziness as in the baseline condition.

## Discussion

Herein we demonstrate that disruption of the inter-hemispheric parietal balance through bilateral application of tDCS (right-anodal/left-cathodal) results in the asymmetrical modulation of the VOR, such that the VOR is suppressed following the right caloric irrigation. In order to delineate the “active electrode” during the bilateral condition we applied unilateral tDCS stimulation. Cathodal stimulation of the left parietal cortex alone resulted in bilateral albeit asymmetrical reduction in VOR slow phase velocities (i.e. greater reduction for the right compared to the left caloric).

The VOR is an adaptable reflex as it is possible to bi-directionally modify its parameters by visual input, mental imagery and habituation to repeated vestibular stimulation impacting upon the central velocity storage mechanism [11]. We rule out the possibility that the modulation of the VOR observed was due to the tDCS impacting upon vestibular afferents, via galvanic labyrinthine stimulation [12], since we observed an asymmetrical VOR modulation. Furthermore, galvanic stimulation induces predominantly torsional vestibular-ocular responses [13], [14] in contrast to the horizontal VOR effects reported here with. Moreover, the asymmetric nature of the VOR modulation observed, here with tDCS and in a previous behavioral experiment [8], suggests that higher order mechanisms may be responsible for the apparent down-regulation.

**Figure 3:**
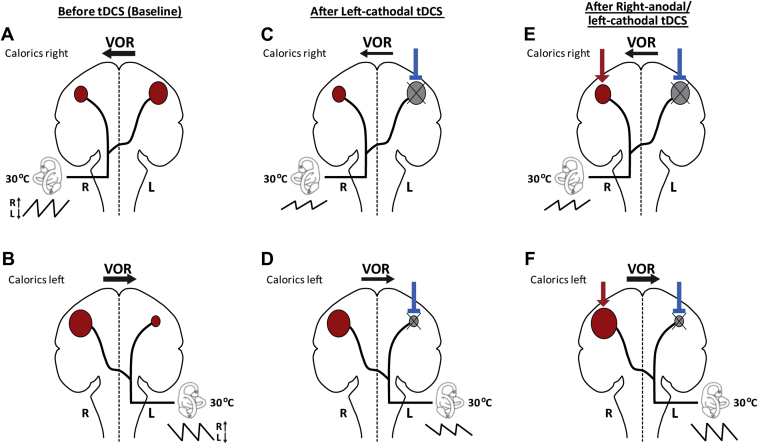
Schematic model of vestibular processing during baseline cold caloric irrigation (A, B) and hypothesized modulation of VOR response after left-cathodal (C, D) and right-anodal/left-cathodal (E, F) tDCS stimulation. The intensity of the cortical activation is represented by the size of the red areas. The direction of slow phase eye movement generated by caloric irrigation is shown by black arrows with the thickness of the arrows indicating eye velocity magnitude. The VOR response following a caloric is exemplified by a short nystagmic trace (inserted below the semi-circular canals), the relative amplitude of which reflects the changes in mean SPV observed. A and B. In the baseline condition, activation of semi-circular canals by caloric irrigation results in stronger projections to contralateral parietal cortex with right hemisphere dominance [1], [7], [15], [16], [17]. C, D, E, and F. Left-cathodal stimulation results in inhibition of the underlying cortex as represented by blue blunt arrow and gray areas; Right-anodal stimulation causes facilitation of the underlying cortex as represented by red arrow and expansion of red areas. C and D. After unilateral left-cathodal inhibitory stimulation, the left parietal lobe may have insufficient resources to process right and left caloric responses, resulting in the bilateral but asymmetric reduction of the slow phase eye velocity. E and F. After bilateral left-cathodal/right-anodal tDCS stimulation, right caloric is insufficiently processed due to inhibition of the left parietal lobe. Left caloric is sufficiently processed with normal VOR response since application of the right-anodal stimulation may compensate for the lack of processing resources in the left parietal lobe. (For interpretation of the references to color in this figure legend, the reader is referred to the web version of this article.)

Previous neuroimaging studies suggest that the insular-parietal cortical regions are implicated in processing vestibular signals [1], [7], [15], [16], [17]. Cold water caloric irrigation appears to result in bilateral activation of the cortical areas however with a preponderance of activation of the contralateral parietal cortex to the side of the irrigated ear [1], [7], [15], [16], [17]. Moreover, right hemisphere dominance in right-handed individuals has been suggested for vestibular cortical processing [1]. Thus, in our baseline pre-tDCS VOR measurements, the cortical processing of the right ear caloric takes place predominately in the left hemisphere with some in the right hemisphere. For left ear caloric the vast majority is processed in the right hemisphere (due to the right hemisphere dominance) with little processing on the left (Fig. 3A and B).

Both bilateral right-anodal/left-cathodal and unilateral left-cathodal tDCS stimulation caused reduction of VOR rightward slow phase velocity following right caloric irrigation. This suggests that cathodal stimulation over the left parietal cortex is the “active” electrode that induces the observed VOR modulation. We suggest that left cathodal stimulation inhibits the left parietal lobe and renders it less able to process the vestibular signals from the right caloric resulting in VOR suppression (Fig. 3C and E). Interestingly, for left ear caloric, the “active” electrode produced differential effects in the bilateral compared to unilateral stimulation conditions. In unilateral left-cathodal condition (Fig. 3D), a reduction in the VOR for left caloric was observed, albeit the reduction was significantly smaller to the one observed for right caloric (Fig. 3C). However, in the bilateral right-anodal/left-cathodal condition, left caloric produced normal VOR responses, which were similar to the responses observed pre-tDCS (Fig. 3F). We propose that the introduction of anodal stimulation over the right hemisphere in the bilateral right-anodal/left-cathodal condition results in a facilitatory effect increasing the processing capacity of vestibular information in the right parietal cortex. Thus, the right hemisphere is able to compensate for the loss of processing power in the left hemisphere and normal VOR response is observed (Fig. 3).

No modulatory effect upon the VOR was observed with any of the other tDCS stimulation conditions. Lack of a modulatory effect in right-cathodal condition is somewhat surprising since inhibition of right parietal cortex should hypothetically result in disruption of inter-hemispheric parietal balance and thus result in alteration of vestibular cortical processing. We propose that the right hemisphere may be able to compensate better than the left hemisphere for the induced inhibition via cathodal tDCS stimulation, thanks to its superior preponderance for vestibular processing [1]. Moreover, an alternative but not mutually exclusive explanation is the proposed functional asymmetry between the two parietal cortices. For visuo-spatial abilities, the right hemisphere appears to exert a stronger inhibition over the left hemisphere suggesting that parietal inter-hemispheric connections are asymmetric [18]. Hence, cathodal tDCS stimulation may cause less inhibitory effect when applied over the right hemisphere compared to the left hemisphere as a result of the functional asymmetry between the parietal cortices.

Our findings are supported by two studies where it was observed that disruption of parietal balance, either via a lesion [2] or possible “overloading” of the right hemisphere [8], resulted in suppressed VOR time constants for leftward chair rotation (rightward slow phase nystagmus). However, no changes in VOR gain were reported in either study [2], [8] which might mean that parietal disruption could down-regulate the central velocity storage mechanism without affecting the VOR gain [8].

However, a more parsimonious explanation for why we now observe a modulation of slow phase velocity but not the duration of the response is most likely related to critical differences in the vestibular stimuli applied in these studies. The rotational stimulus previously used lasts <1 s (velocity steps) whereas the temperature gradient created by caloric irrigation lasts several minutes [19]. Slow phase velocity during step velocity rotations, as employed in the two previous studies where a modulation of the VOR time constant (but not gain) was observed, is measured during the high frequency component of the rotational stimulus – typically within 1–2 s of the high acceleration delivered. At this point, the slow acting velocity storage mechanism is not involved. In the present study, a caloric stimulus was deployed and the peak velocities reported are reached 60–80 s after stimulus onset (see Figs. 1 and 2), almost certainly under the influence of the velocity storage integrator. Thus, disruption of parietal inter-hemispheric balance, either via a right hemisphere lesion [2], possible behavioral overloading of the non-dominant hemisphere [8] or by DC neuromodulation (as in this study), can result in down-regulation of the maximal slow phase VOR velocities by affecting the time constant or gain components of the central velocity storage mechanism.

Very recently [14], a tDCS experiment induced increases in rotational vestibular thresholds (vestibulo-perceptual and vestibular-ocular), albeit bilaterally and symmetrically. The difference with the clear asymmetries observed in the current experiment may be due to several factors: caloric stimuli engage only one ear (and its ascending projections) at a time; threshold and suprathreshold responses are known to differ drastically [21], and furthermore threshold responses are unlikely to recruit velocity storage pathways. Of note, these findings could have potential clinical implications. Indeed, it has recently been proposed that TMS may be applied to treat visuo-spatial attentional disorders [20]. We report that tDCS can modulate low-level brainstem function (i.e. VOR) signifying potential applications to address VOR and vestibulo-perceptual asymmetries in peripheral vestibular disorders such as acute vestibular neuritis [21].

In summary, our results provide novel evidence using neuromodulation that disruption of parietal balance via tDCS inhibition of the left hemisphere in right-handed subjects results in an asymmetrical suppression of the VOR. Our results provide support for the presence of functional asymmetry between the two parietal lobes and imply right hemisphere dominance for vestibular cortical processing.
